# Dicer Regulates Differentiation and Viability during Mouse Pancreatic Cancer Initiation

**DOI:** 10.1371/journal.pone.0095486

**Published:** 2014-05-01

**Authors:** John P. Morris, Renee Greer, Holger A. Russ, Guido von Figura, Grace E. Kim, Anke Busch, Jonghyeob Lee, Klemens J. Hertel, Seung Kim, Michael Mcmanus, Matthias Hebrok

**Affiliations:** 1 Diabetes Center, Department of Medicine, University of California San Francisco, San Francisco, California, United States of America; 2 Department of Pathology, University of California San Francisco, San Francisco, California, United States of America; 3 Department of Microbiology and Molecular Genetics, University of California Irvine, Irvine, California, United States of America; 4 Department of Developmental Biology, Howard Hughes Medical Institute, Stanford University School of Medicine, Stanford, California, United States of America; Indiana University School of Medicine, United States of America

## Abstract

miRNA levels are altered in pancreatic ductal adenocarcinoma (PDA), the most common and lethal pancreatic malignancy, and intact miRNA processing is essential for lineage specification during pancreatic development. However, the role of miRNA processing in PDA has not been explored. Here we study the role of miRNA biogenesis in PDA development by deleting the miRNA processing enzyme Dicer in a PDA mouse model driven by oncogenic Kras. We find that loss of Dicer accelerates Kras driven acinar dedifferentiation and acinar to ductal metaplasia (ADM), a process that has been shown to precede and promote the specification of PDA precursors. However, unconstrained ADM also displays high levels of apoptosis. Dicer loss does not accelerate development of Kras driven PDA precursors or PDA, but surprisingly, we observe that mouse PDA can develop without Dicer, although at the expense of proliferative capacity. Our data suggest that intact miRNA processing is involved in both constraining pro-tumorigenic changes in pancreatic differentiation as well as maintaining viability during PDA initiation.

## Introduction

Pancreatic ductal adenocarcinoma (PDA) appears to develop through a series of ductal precursor lesions, including the most common type, pancreatic intraepithelial neoplasia (PanIN). Both PanINs and PDA exhibit Kras mutations, which may be an initiating event. Mouse models support this notion as targeted and persistent expression of mutant Kras in the mouse pancreatic epithelium both recapitulates the PanIN to PDA sequence observed in humans, and is required for disease maintenance [1], [2], [3]. Evidence from mouse models suggests that mutant Kras can contribute to PDA initiation by reprogramming acinar cells into a duct like lineage capable of becoming PanINs via a process termed acinar to ductal metaplasia (ADM) [4], [5], [6]. This ability to change pancreatic plasticity may be an important step in PDA initiation, as acinar cells have been shown to be dramatically more sensitive to Kras dependent PanIN development compared to duct cells [7]. Since attempts at direct inhibition of oncogenic Kras have been generally unsuccessful[8], defining critical mediators of pro-tumorigenic differentiation and viability downstream of mutant Kras may represent alternative therapeutic approaches.

MicroRNAs (miRNAs) are a class of small, non-coding RNAs that regulate gene expression post-transcriptionally. Aberrant miRNA levels are associated with tumor development, and lead to inappropriate expression of oncogenes and tumor suppressors[9]. Cancer associated miRNA levels result from the misexpression of specific miRNAs as well as deregulation of the miRNA biogenesis pathway[10]. miRNAs are generally downregulated in human cancers[11], and inhibited miRNA processing promotes tumorigenesis[12]. However, loss of miRNA processing may be incompatible with the development of some tumors[13], [14], [15], suggesting that critical thresholds of miRNA processing may be involved in maintaining viability during transformation. While miRNAs are misexpressed in PanINs and PDA, and are known to regulate pathways that contribute to PDA initiation and progression[16], [17], [18], [19], the role that miRNA processing plays in PDA development has not been investigated.

By deleting the miRNA-processing enzyme Dicer in a Kras driven mouse model of PDA, we find that miRNA processing regulates both differentiation and viability during Kras driven pancreatic transformation. Dicer deletion promotes Kras driven loss of acinar identity and ADM but also results in increased levels of apoptosis and decreased expression of genes implicated in maintaining viability during PanIN development and in PDA. Surprisingly, we find that mouse PDA can develop in the absence of Dicer, although with decreased proliferation. Taken together, this work suggests a critical role for miRNA processing in Kras driven PDA initiation.

## Results

### Dicer Loss Accelerates Kras Driven Ductal Metaplasia

Dicer deletion in early pancreatic progenitors results in pancreatic agenesis and post-natal death [20]. Therefore, we tested if a distinct Pdx1 driven Cre strain, *Pdx1-Cre^Late^*, permitted pancreatic development in the setting of loss of Dicer function. *Pdx1-Cre^Late^* results in delayed developmental activity compared to the Pdx1-Cre driver employed by Lynn *et al*, resulting in recombination in a more restricted set of adult cells, specifically most acini, some endocrine cells, and rarely in duct cells[21]. Suggesting temporal requirements for Dicer in pancreatic development, *Pdx1-Cre^Late^; Dicer^flox/flox^* (*Dicer^Homo^*) mice thrived and displayed grossly normal pancreatic development at p0 even in the context of significantly decreased Dicer expression compared to control *Pdx1-Cre^Late^; Dicer^flox/+^* mice (*Dicer^Het^*) (Figure S1 A,B,E).

**Figure 1:**
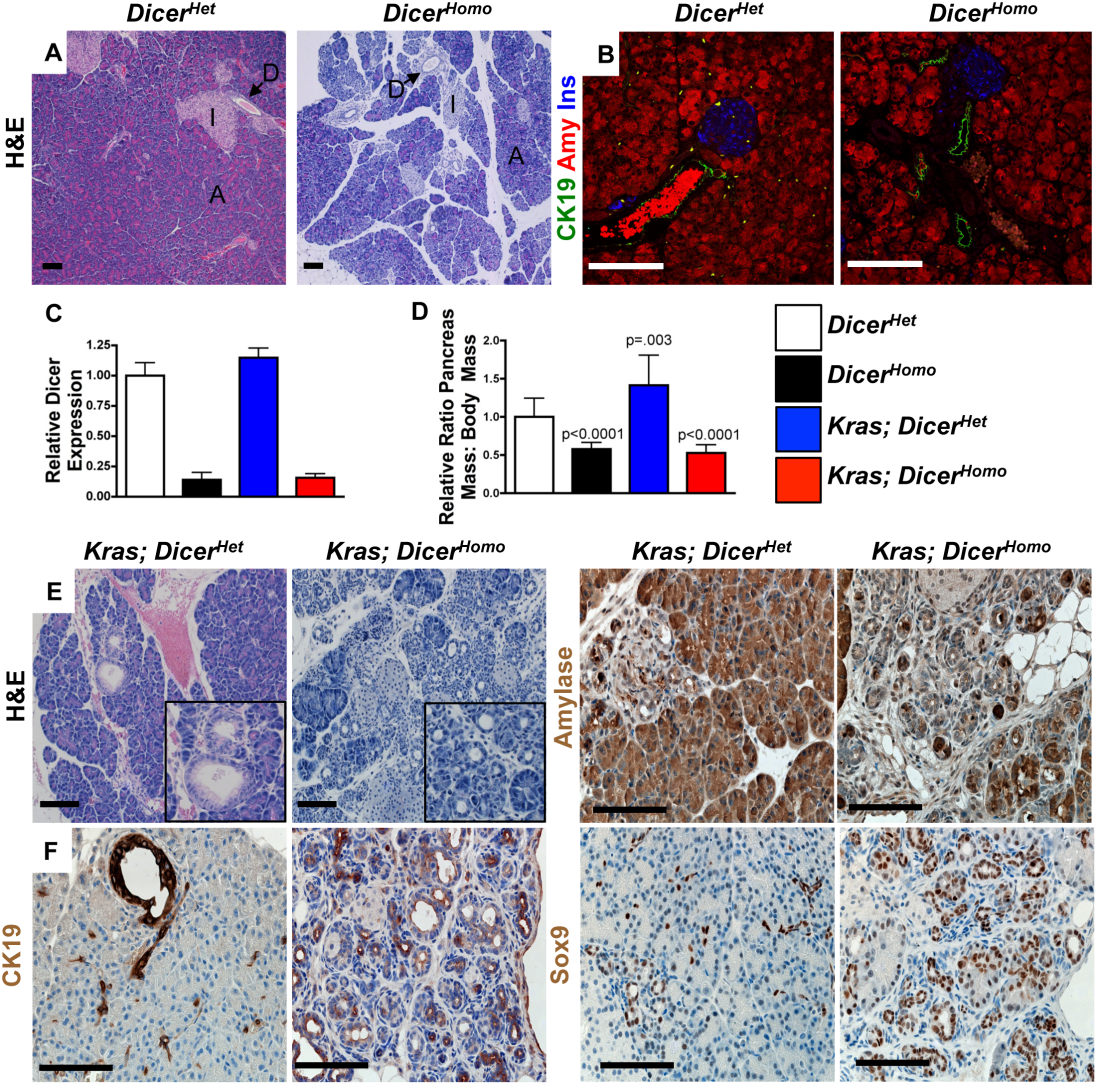
Dicer loss accelerates Kras driven ADM. (**A**)**.** Hematoxylin and Eosin (H&E) staining of 3 week old pancreas from *Dicer^Het^* and *Dicer^Homo^* mice. (A) acini, (D) duct, and (I) islets. Scale bar 100 µM. (**B**) Immunofluorescence for amylase (red), CK19 (green), and insulin (blue) in 3 week old *Dicer^Het^* and *Dicer^Homo^* mice. Scale bar 100 µM. (**C**)**.** Reduced Dicer expression at 3 weeks in RNA extracted from pancreas tissue of indicated genotypes. Mean ± SD, n = 3. (**D**)**.** Pancreas mass: body weight ratio at 3 weeks in the indicated genotypes. P-values are calculated from two-tailed, unpaired t-tests comparing *Dicer^Het^* and indicated genotypes. (Mean ± SD, *Dicer^Het^* n = 17, *Dicer^Homo^* n = 14, *Kras; Dicer^Het^ n = 9, Kras; Dicer^Homo^* n = 14). (**E**)**.** H&E staining of 3 week old *Kras; Dicer^Het^* and *Kras; Dicer^Homo^* mice. Insets: E-left, Rare focus of metaplasia and PanIN; right, ductal metaplasia in *Kras; Dicer^Homo^* mice. Scale bar 100 µM. (**F**) Immunohistochemistry for Amylase, Sox9, CK19 in 3 weeks old *Kras; Dicer^Het^* and *Kras; Dicer^Homo^* mice. Scale bar 100 µM.

At 3 weeks of age, acini, ducts, and islets were recognizable in *Dicer^Homo^* mice (Figure 1A), although there were some exocrine areas with decreased eosin staining. Immunofluorescence revealed normal distribution of the acinar marker amylase, duct marker CK19, and β-cell marker insulin (Figure 1B). Exocrine morphology was disturbed though, as we often found disorganized acini with small, fragmented cells not detected in controls (Figure 1B, Figure S2), similar to disorganized acinar cells observed in somatic Dicer hypomorphs[22]. Pancreatic mass in *Dicer^Homo^* mice was also reduced (Figure 1D) in the setting of decreased Dicer expression (Figure 1C). Despite the changes in morphology, we did not observe cells co-expressing amylase and elevated levels of CK19 as observed in acinar cells undergoing Kras driven ADM (Figure S3), suggesting that the disorganized acinar cells were not actively undergoing ADM.

To test the effect of Dicer loss on Kras driven pancreatic transformation, we generated *Pdx1-Cre^Late^; LSL-Kras^G12D^; Dicer^flox/flox^* (*Kras; Dicer^Homo^*) mice. Like other models targeting mutant Kras to the embryonic pancreas, *Pdx1-Cre^Late^; LSL-Kras^G12D^* mice gradually develop ADM as well as PanINs, with PDA arising in some animals after long latency[23]. Similar to such models[1], at 3 weeks of age, pancreas mass in *Pdx1-Cre^Late^; LSL-Kras^G12D^; Dicer^flox/+^* (*Kras; Dicer^Het^*) mice was slightly, but significantly, increased compared to *Dicer^Het^* mice (Figure 1D). The exocrine compartment in *Kras; Dicer^Het^* mice appeared grossly normal, predominantly composed of amylase positive acinar cells, with rare areas of amylase negative ADM and low grade PanINs expressing moderate to high levels of CK19 and Sox9 (Figure 1F), ductal/pancreatic embryonic progenitor markers characteristic of Kras driven ADM and PanIN formation[6]. In contrast, *Kras; Dicer^Homo^* pancreata displayed decreased Dicer expression (Figure 1C), were atrophic (Figure 1D), and possessed widespread replacement of the exocrine compartment with metaplastic duct structures resembling rare ADM in *Kras; Dicer^Het^* mice (Figure 1E). Ductal metaplasia in *Kras; Dicer^Homo^* mice displayed decreased expression of amylase, low to moderate expression of CK19, and strong expression of Sox9 (Figure 1F). Sox9 accumulation was also observed in structures retaining some acinar morphology (Figure 1F). Ductal metaplasia was not observed at p0 in *Kras; Dicer^Homo^* mice (Figure S1D), suggesting that Dicer deficiency in the context of mutant Kras does not block embryonic acinar development, and ADM occurs between birth and 3 weeks of age. Therefore, Dicer loss in the context of mutant Kras dramatically accelerates ADM, a process that has been shown to both precede and promote Kras driven PanIN formation[4], [6], [24].

### Dicer Loss Compromises Acinar Identity and Promotes Kras Driven Acinar to Ductal Reprogramming

**Figure 2:**
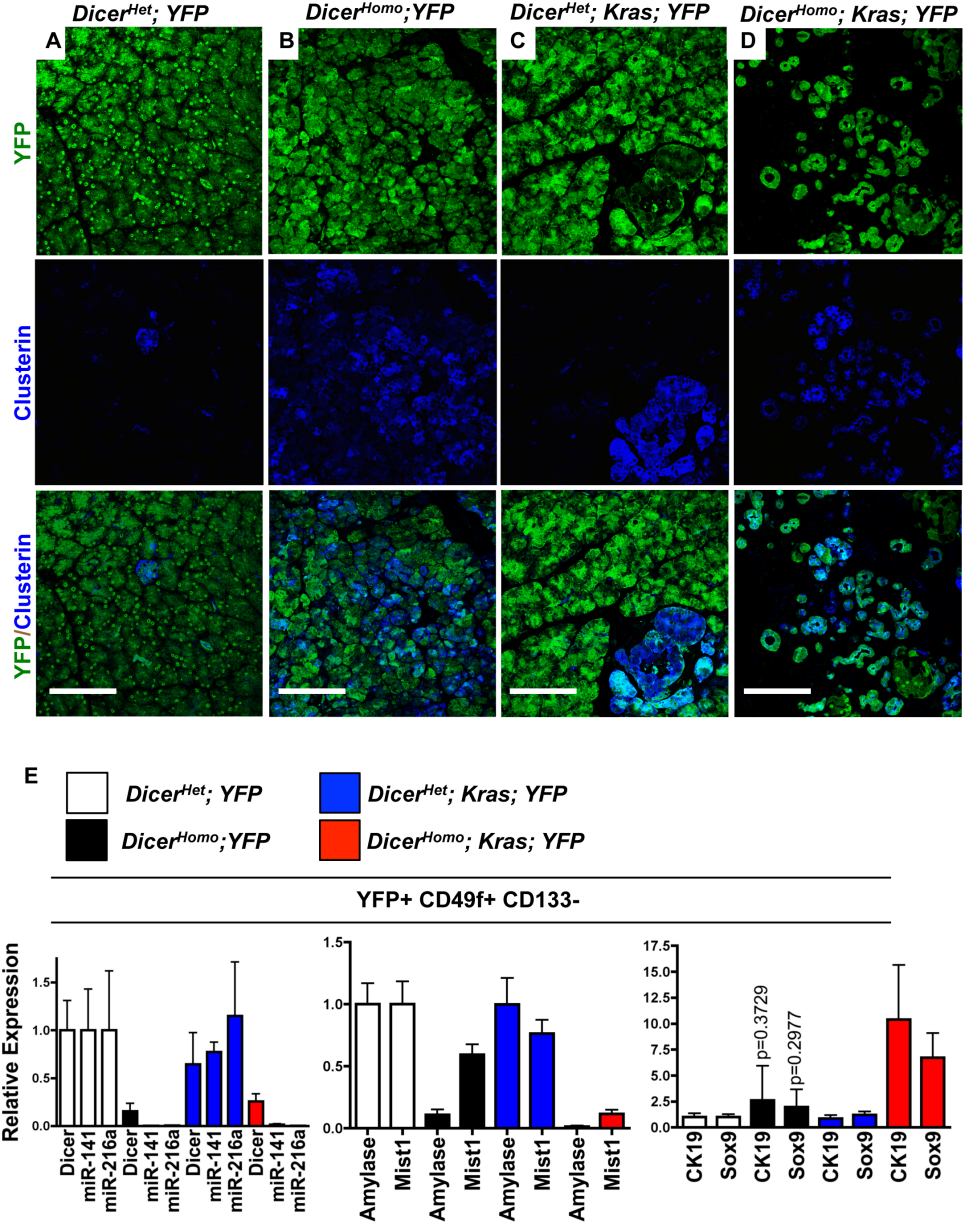
Dicer loss promotes Kras driven loss of acinar identity and ductal reprogramming. (**A–D**) Clusterin (blue) and YFP (green) staining in 3 week old *Dicer^Het^; YFP* (A), *Dicer^Homo^; YFP* (B), *Kras; Dicer^Het^; YFP* (C), and *Kras; Dicer^Homo^; YFP* mice (D). Scale bar 100 µM (**E**) RT-PCR analysis of Dicer, miRNA-141 and 216b (left), acinar enriched genes Amylase and Mist1 (center), and duct enriched genes CK19 and Sox9 (right) of YFP+, CD49f+, CD133- cells from *Dicer^Het^; YFP* (n = 4), *Dicer^Homo^; YFP* (n = 4), *Kras; Dicer^Het^; YFP* (n = 3), and *Kras; Dicer^Homo^; YFP* (n = 3) mice. Mean ± SD. P-values are calculated from two-tailed, unpaired t-tests comparing *Dicer^Het^* and *Dicer^Hom^°*CK19 and Sox9 expression values.

To explore why *Kras; Dicer^Homo^* mice undergo accelerated Kras driven ADM, we asked if Dicer deficient acinar cells inappropriately display properties associated with ADM, such as markers of acinar stress and loss of acinar identity. To focus on cells that had undergone Cre recombination we included a Cre inducible YFP allele expressed conditionally from the Rosa26 locus (*R26-EYFP*). YFP staining revealed that both the exocrine compartment in *Pdx1-Cre^Late^; Dicer^flox/flox^; R26-EYFP* (*Dicer^Homo^; YFP*) mice and ADM in *Pdx1-Cre^Late^; LSL-Kras^G12D^; Dicer^flox/flox^; R26-EYFP* (*Kras; Dicer^Homo^; YFP*) mice derived from cells in which Cre was active, and not from expansion of an un-recombined population (Figure 2B, D).

To determine if Dicer loss led to acinar stress associated with ADM, we examined expression of clusterin, a marker of stressed, de-differentiated acini[6], [25]. Clusterin was mainly absent in YFP+ acinar cells of control *Dicer^Het^; YFP* and *Kras; Dicer^Het^; YFP* mice, but was present in rare YFP+ ADM in *Kras; Dicer^Het^; YFP* animals (Figure 2A, C). Suggesting that Dicer deficient cells are under stress observed in ADM, clusterin was not only uniformly observed in YFP+ ADM in *Kras; Dicer^Homo^; YFP* mice, but also widely expressed in YFP+ acinar cells in *Dicer^Homo^; YFP* animals (Figure 2B, D).

To test if acinar stress corresponded with decreased acinar identity, we modified a sorting protocol previously developed for isolation of pancreatic progenitors[26] to specifically separate differentiated acinar and ductal cells. We found that in the adult pancreas, adult acini and ducts express the epithelial marker CD49f, while differentiated ducts also express CD133 (Figure S4B, C). RT-PCR for *Amylase* and *Mist1*, and *CK19* and *Sox9*, enriched in acinar and duct cells, respectively, revealed that sorting based on CD49f and CD133 allowed for efficient separation of these populations (Figure S4A, 4D). Expression of Notch effectors Hes1, Hey1, and Hey2, restricted to centroacinar and terminal duct cells in the adult pancreas[27], appeared to segregate with the CD49f+CD133+ duct population (Figure S4E).

YFP+, CD49f+CD133- cells were sorted from all 4 genotypes. At 3 weeks of age Dicer levels and expression of 2 abundantly expressed miRNAs were considerably reduced in cells sorted from *Dicer^Homo^; YFP* and *Kras; Dicer^Homo^; YFP* mice compared to controls with and without Kras (Figure 2E left). To determine the effect of Dicer loss on exocrine differentiation, we analyzed expression of the acinar markers Amylase and Mist1 and the duct markers CK19 and Sox9, in YFP+, CD49f+CD133- cells. Sorted cells from *Dicer^Homo^; YFP* mice displayed considerably decreased expression of *Amylase* and modestly decreased *Mist1* compared to cells from *Dicer^Het^; YFP* mice (Figure 2E middle). Although expression of markers of acinar differentiation were reduced, we did not observe a consistent increase in the ductal markers *CK19* or *Sox9* in these cells (Figure 2E right), similar to the lack of widespread CK19 misexpression noted in Figure 1D. In CD49f+CD133- cells from *Kras; Dicer^Homo^; YFP* mice however, *Amylase* and *Mist1* expression was further decreased, and *CK19* and *Sox9* expression was increased, compared to cells from all other genotypes, consistent with histological increase in ADM.

Interestingly, we also observed a modest, but not statistically significant, trend toward reduction in Mist1 expression in cells sorted from *Kras; Dicer^Het^; YFP* mice compared to *Dicer^Het^; YFP* controls, suggesting that mutant Kras alone may begin compromising acinar identity before the induction of ductal genes or morphology. Taken together, this data suggests that Dicer loss compromises acinar identity and induces a stressed state that accelerates Kras driven ductal reprogramming and ADM.

### Dicer Loss does not Accelerate PanIN or PDA Development

**Figure 3:**
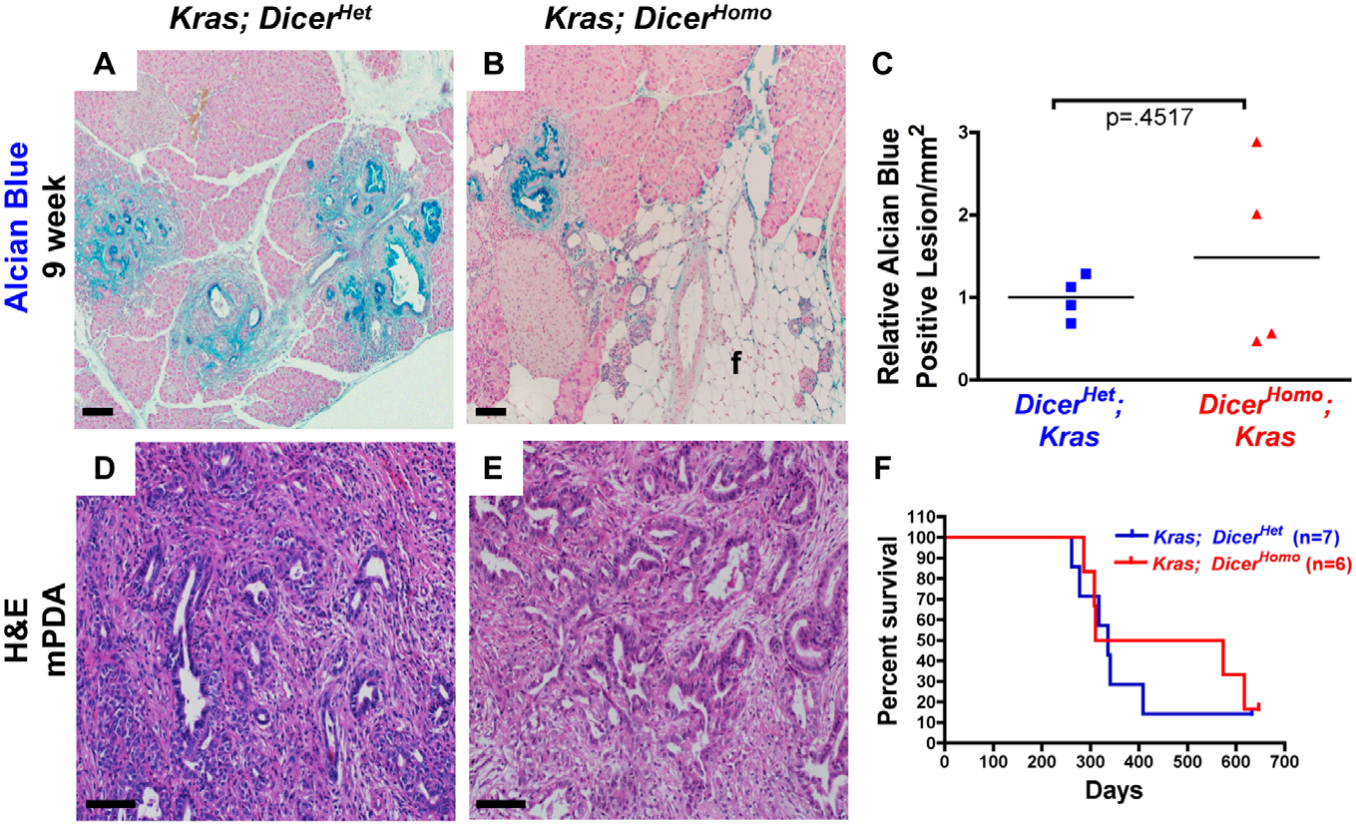
Kras driven PanIN and PDA progression in Dicer conditional mice. (**A–B**) Representative Alcian Blue staining at 9 weeks in *Kras; Dicer^Het^* (A) and *Kras; Dicer^Homo^* mice (B). f: fat. Scale bar 100 µM. **C**, Quantification of alcian blue positive lesions of 9 weeks old *Kras; Dicer^Het^* and *Kras; Dicer^Homo^* mice. Lines indicate means. P-value calculated from a two-tailed, unpaired t-test. (**D,E**) Representative histology of PDA from *Kras; Dicer^Het^*(D) and *Kras; Dicer^Homo^* mice (E). Scale bar 100 µM. (**F**) Survival curve of *Kras; Dicer^Het^* (n = 7) and *Kras; Dicer^Homo^* (n = 6) mice.

Loss of acinar identity and ADM accelerates PanIN development [6], [24], [28], [29] and accelerated ADM and PanIN development, induced by acute and chronic pancreatitis for example, can result in decreased PDA latency [30], [31], [32]. Therefore, we expected to observe faster PanIN and PDA development in *Kras; Dicer^Homo^* mice compared to control *Kras; Dicer^Het^* mice. Despite the dramatic difference in ADM observed at 3 weeks in *Kras; Dicer^Homo^* compared to *Kras; Dicer^Het^* mice, quantification of PanIN lesions in a small cohort of mice at 9 weeks revealed no statistically significant difference in the frequency of Alcian Blue positive ductal lesions, suggesting that Dicer loss does not accelerate PanIN development (Figure 3A, B, C). Long-term disease progression also converged independent of conditional Dicer status. We found no statistically significant difference in survival of a small cohort of aged *Kras; Dicer^Homo^* and *Kras; Dicer^Het^* mice (Median survival 336 versus 441.5 days, χ^2^ = .2271, p = 0.6637, calculated using the Logrank test) (Figure 3F). 6/7 of *Kras; Dicer^Het^* mice developed locally or widely invasive PDA that was generally moderately differentiated, but included one undifferentiated cancer. 4 out of 6 *Kras; Dicer^Homo^* mice developed moderately differentiated, locally or widely invasive, PDA with similar frequency of metastasis and cellular morphology (Figure 3D, E, Table S1).

Dicer deleted cells have been shown to be outcompeted by unrecombined cells in other endoderm derived organs, in the liver for example [33], and elimination of recombined cells could contribute to the lack of expected difference in disease progression in *Kras; Dicer^Homo^* versus *Kras; Dicer^Het^* mice. In contrast to the 3 week time point when normal acinar cells were much less frequent in *Kras; Dicer^Homo^* versus *Kras; Dicer^Het^* mice, considerable areas of normal acinar tissue were found in Kras; Dicer^H*o*m*o*^ mice at this time point (Figure 3A, B). We also noted the presence of fat replacing pancreas tissue in some *Kras; Dicer^Homo^* animals (Figure 3B). Examination of tissue YFP distribution in the pancreas of *Kras; Dicer^Het^; YFP* versus *Kras; Dicer^Homo^; YFP* animals revealed a notable decrease in YFP distribution at 9 weeks (Figure S5), suggesting repopulation with cells that had not undergone recombination. Therefore, despite initially promoting ADM that normally accelerates PanIN and PDA development, PDA progression is not substantially enhanced in the absence of Dicer and may be associated with selection against Dicer deficiency.

### Dicer Loss Compromises Viability during Kras Driven ADM

**Figure 4:**
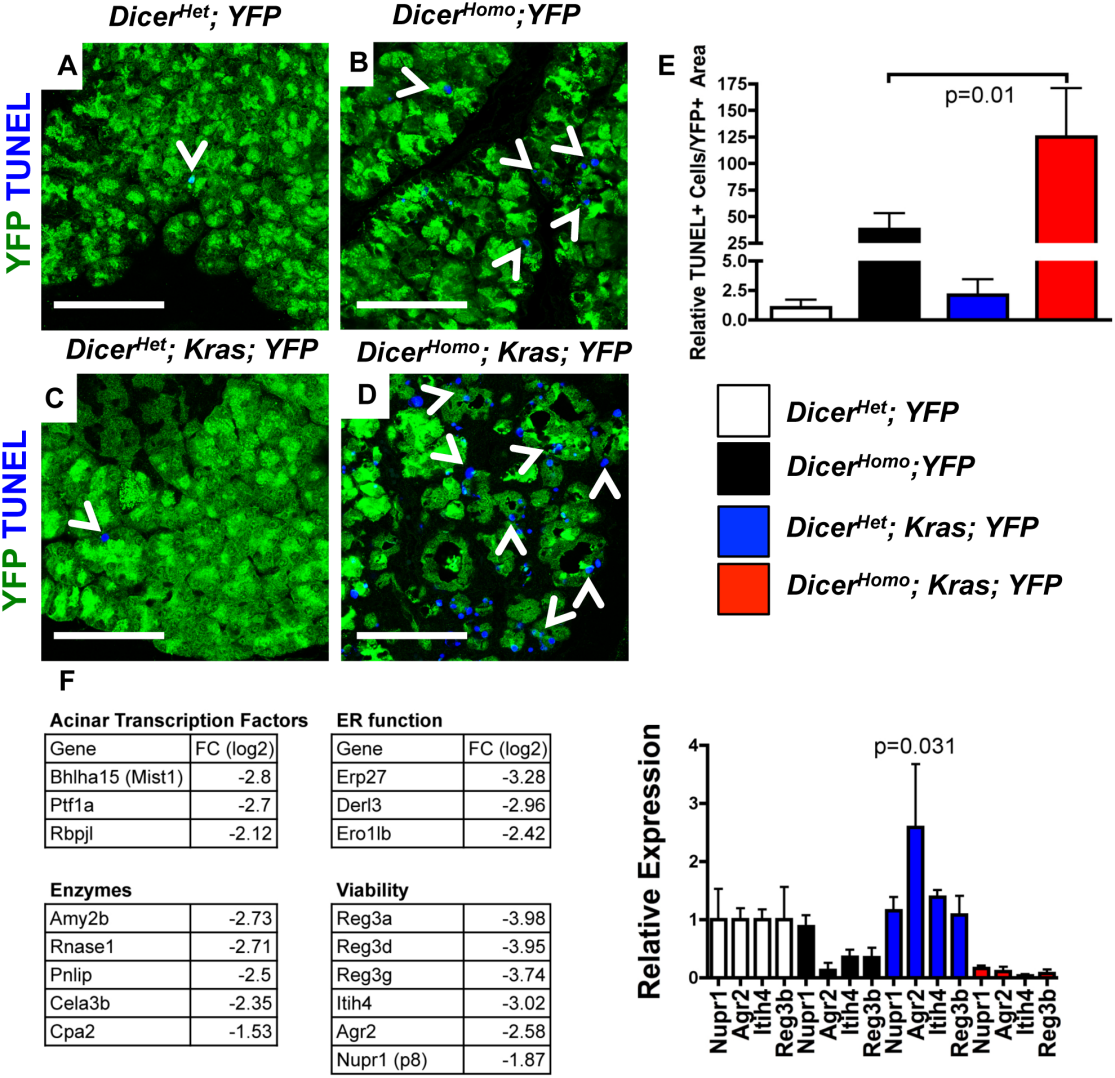
Dicer loss compromises viability during unconstrained Kras driven ADM. (**A–D**) TUNEL (blue) and YFP (green) staining in 3 weeks old *Dicer^Het^; YFP* (A), *Dicer^Homo^; YFP* (B), *Kras; Dicer^Het^; YFP* (C), and *Kras; Dicer^Homo^; YFP* mice (D). Scale bar 100 µM. **E**. Quantification of TUNEL+YFP+ cells in (A-D). Mean ± SD. *Dicer^Het^; YFP* (n = 3), *Dicer^Homo^; YFP* (n = 4), *Kras; Dicer^Het^; YFP* (n = 3), and *Kras; Dicer^Homo^; YFP* mice (n = 4). P-value calculated from a two-tailed, unpaired t-test of TUNEL+YFP+ cells in *Dicer^Homo^; YFP* and *Kras; Dicer^Homo^; YFP* mice. **F.** Acinar enriched and viability associated genes from expression analysis (left). RT-PCR analysis of Nupr1, Agr2, Itih4, and Reg3b in acinar enriched cells from *Dicer^Het^; YFP* (n = 4), *Dicer^Homo^; YFP* (n = 4), *Kras; Dicer^Het^; YFP* (n = 3), and *Kras; Dicer^Homo^; YFP* (n = 3) mice (right). Mean ± SD. P-value calculated from a two-tailed, unpaired t-test comparing Agr2 expression in sorted cells from *Dicer^Het^; YFP* and *Kras; Dicer^Het^; YFP* mice.

Dicer loss leads to increased apoptosis in a number of endodermal organs (e.g. liver and intestine [33], [34]. To address if the lack of predicted acceleration of PanIN development in *Kras; Dicer^Homo^* mice might involve increased cell death, we audited TUNEL+/YFP+ cells at 3 weeks of age. In contrast to the rare double positive cells in *Dicer^Het^; YFP* and *Kras; Dicer^Het^; YFP* mice, *Dicer^Homo^; YFP* mice displayed a higher relative rate of TUNEL+/YFP+ cells (Figure 4B, E). Furthermore, we found an additional increase in double positive cells in *Kras; Dicer^Homo^; YFP* mice compared to *Dicer^Het^; YFP* and *Kras; Dicer^Het^; YFP* mice(Figure 4D, E). Interestingly, Dicer loss appeared to synergize with mutant Kras to promote cell death, as *Kras; Dicer^Homo^; YFP* mice exhibited significantly more apoptosis than *Dicer^Homo^; YFP* cells (Figure 4E). Therefore, while intact Dicer processing constrains Kras driven acinar to ductal reprogramming, some Dicer dependent signals may be required for maintaining viability during Kras driven metaplasia.

We performed expression analysis to screen for Dicer dependent factors potentially involved in maintaining viability during Kras driven ADM. To better synchronize ADM in *Kras; Dicer^Het^* and *Kras; Dicer^Homo^* mice, we utilized caerulein induced pancreatitis. As predicted from previous studies[6], caerulein treatment in control, 3 weeks old *Kras; Dicer^Het^* mice led to the development of duct like structures 2 days after treatment (Figure S6A), and widespread replacement of the exocrine compartment with ADM, PanINs, and fibrosis, 21 days after treatment (Figure S6C). Mirroring the lack of accelerated PanIN development between 3 and 9 weeks in *Kras; Dicer^Homo^* mice (Figure 3F), we observed less metaplasia and considerably more normal acinar tissue 21 days following caerulein treatment in *Kras; Dicer^Homo^* mice (Figure S6D). RNA-Seq Analysis of pooled RNA from FACS isolated YFP+, CD49f+, CD133- acinar cells from 3 weeks old *Kras; Dicer^Het^; YFP* (3 mice) and *Kras; Dicer^Homo^; YFP* (5 mice) mice 2 days after caerulein (Figure S6E) revealed ∼120 genes significantly downregulated between mutant and control. Supporting the observation that loss of Dicer compromises acinar differentiation, 42 genes associated with terminally differentiated acinar cells were significantly downregulated (Figure 4F, Table S2, complete data set Table S3). Also reduced were genes implicated in maintaining viability during regeneration or tumorigenesis, including Nupr1/p8, Agr2, Itih4, and members of the Reg3 protein family (Figure 4F, Table S2). Recently, both Nupr1 and Agr2 have been shown to be overexpressed during PanIN-PDA progression and to play a role in PanIN development and maintaining PDA viability[35], [36], [37]. Reg3 family protein expression is activated in response to pancreatitis[38], and family member Reg3b (also known as Pap1) specifically is implicated in maintaining acinar viability and can act downstream of Nupr1[39], [40]. Although Itih4 has not been implicated in PDA development, it has been shown to be involved in maintaining viability downstream of the critical PDA mediator c-myc[41] in a model of liver cancer[42]. RT-PCR analysis of these candidate genes in sorted acini from 3 week-old mice revealed that their expression inversely correlated with apoptosis (Figure 4F, right). Nupr1, Agr2, Itih4, and Reg3b expression was reduced in sorted cells from *Kras; Dicer^Homo^; YFP* mice compared to control *Dicer^Het^; YFP* and *Kras; Dicer^Het^; YFP* animals. Also, Agr2, Itih4, and Reg3b expression was reduced in sorted cells from *Dicer^Homo^; YFP* mice compared to controls. Interestingly, Agr2 was also significantly increased in cells sorted from *Kras; Dicer^Het^; YFP* mice compared to cells from *Dicer^Het^; YFP* mice, suggesting that Kras may contribute to Agr2 upregulation even before ADM occurs. Therefore, Dicer expression is required for the maintenance of the mature acinar differentiation state and for expression of pro-viability genes during Kras driven ADM.

### Mouse PDA can Develop in the Absence of Dicer

**Figure 5:**
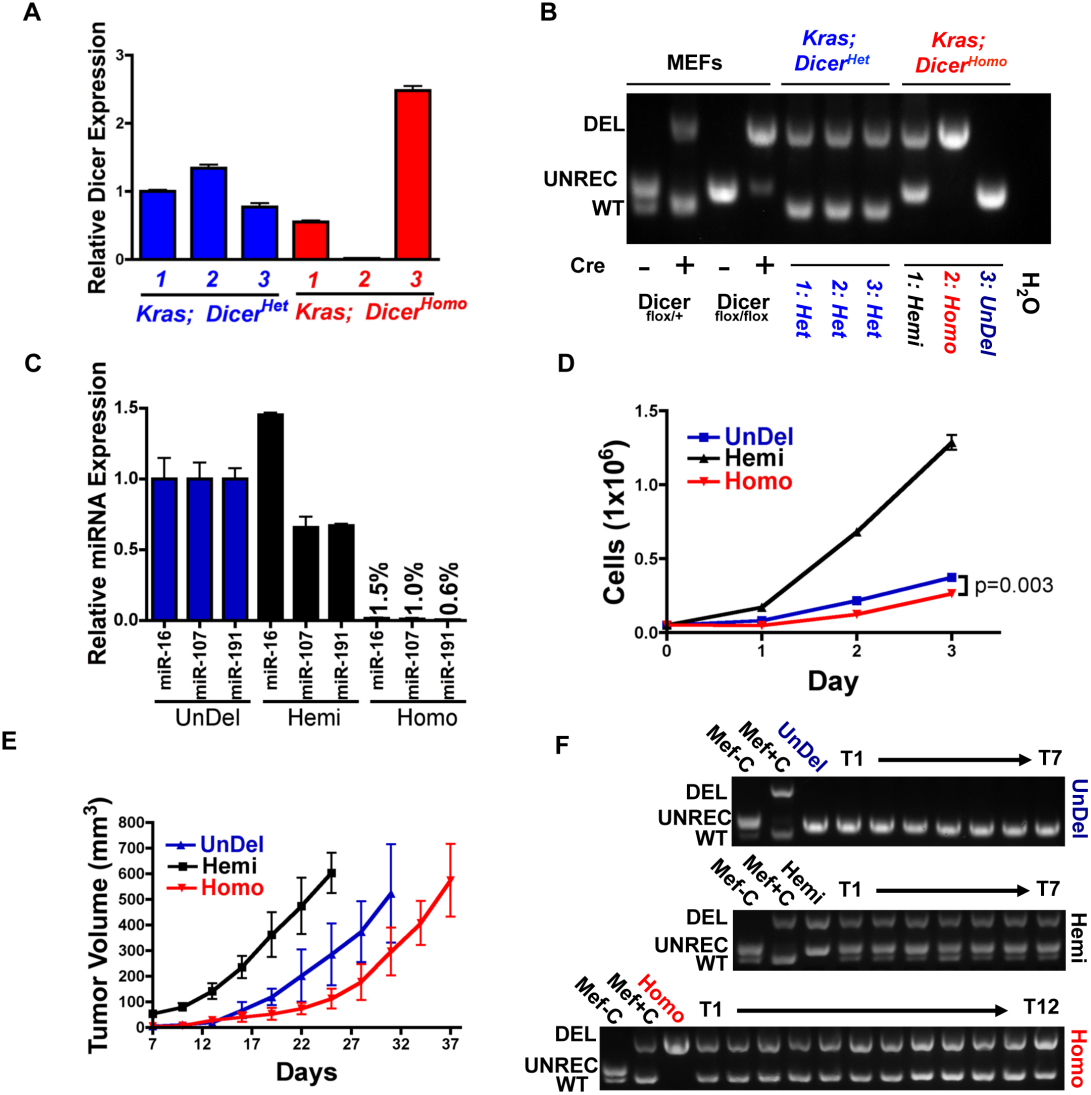
Dicer loss can be tolerated during mouse PDA development. **A.** Dicer RT-PCR in PDA cell lines generated from *Kras; Dicer^Het^* (blue) and *Kras; Dicer^Homo^* (red) mice. Each bar represents data from 3 independent wells of each cell line. Mean ± SD. **B.** PCR detecting WT, unrecombined (UNREC), and deleted (DEL) Dicer genomic locus in cell lines from A. Controls are amplified DNA from mouse embryonic fibroblasts (MEFs) of indicated Dicer genotype following adenoviral Cre treatment. **C.** miRNA QPCR for miRNA-16, 107, 191 in cell lines derived from *Kras; Dicer^Homo^* mice. Each bar represents 3 independent wells of each cell line. Mean ± SD. **D.** Growth of PDA cell lines derived from *Kras; Dicer^Homo^* mice. Each point represents values from 3 independent wells. Mean ± SD. P-value calculated from a two-tailed, unpaired t-test comparing the number of cells at 72 hours in cultures of Undel and Homo cells. **E.** Subcutaneous tumor growth of PDA cell lines derived from *Kras; Dicer^Homo^* mice. Mean ± SD at each timepoint. Undel (n = 7), Hemi (n = 7), Homo (n = 12). **F.** PCR detecting WT, UNREC, and DEL Dicer genomic locus in subcutaneous tumors at time of sacrifice in E. Controls are Dicer^fl*o*x/+^ MEFs treated with adenoviral Cre as in B. Tumor DNAs are preceded by amplification of DNA from parental cell lines.

Dicer levels have been shown to be important regulators of tumorigenesis, acting as a haploinsufficient tumor suppressor in the context of mutant Kras in both lung tumors and sarcomas, as well as in retinoblastoma[13], [14]. However, tumors in these models appear to result from cells that have retained expression of at least one unrecombined conditional allele. To determine if Dicer deficient cells were capable of contributing to Kras driven PDA we tested Dicer expression and genomic recombination from 3 PDA cell lines generated from *Kras; Dicer^Het^* and *Kras; Dicer^Homo^* mice. By RT-PCR we observed similar Dicer expression in all three PDA cell lines derived from *Kras; Dicer^Het^* mice (Figure 5A). Due to the high rate of apoptosis observed at 3 weeks of age, the reduced expression of genes that support PanIN development, and the lack of accelerated disease progression, we expected to observe retention of at least one allele in PDA lines derived from *Kras; Dicer^Homo^* PDA. Surprisingly, we found that one line displayed dramatically decreased Dicer expression, while the two remaining lines expressed either a similar, or higher, level as *Kras; Dicer^Het^* derived lines. Allele specific PCR revealed that Dicer recombination correlated directly with expression (Figure 5B: “Undel”, no conditional Dicer allele recombination; “Hemi”, recombination of one allele; “Homo”, recombination of both alleles). RT-PCR for 3 mature miRNAs reported to be over-expressed in human PDA[43], [44] revealed reduction in all 3 miRNAs in the homozygous deleted line to less than 95% of that expressed in the undeleted line (Figure 5C). Therefore, Kras driven PDA in the mouse appears able to evade negative selection during disease initiation and develop in the absence of Dicer. As predicted from models demonstrating that downregulation, but not complete loss of miRNA processing, can enhance tumorigenesis[12], [13], the hemizygous deleted line grew fastest, while the homozygous deleted line slowest (Figure 5D). All 3 cell lines were also able to form tumors when implanted subcutaneously in immune deficient mice, with kinetics mirroring the cell culture growth rates (Figure 5E). Allele specific PCR revealed that tumors did not result from cells that underwent spontaneous recombination (UnDel, Hemi), or from a contaminating subpopulation of Dicer competent cells (Homo), although we did observe the presence of the WT Dicer amplicon in tumors derived from the Hemi and Homo lines, indicative of host stromal recruitment (Figure 5F). Therefore, although Dicer elimination impairs viability during the earliest stages of Kras driven neoplasia, loss of Dicer is not mutually exclusive with pancreatic transformation.

## Discussion

Dicer function is essential for the development of several organs, including endoderm-derived tissues such as the liver and the gut[33], [34]. Maintenance of Dicer expression in early Pdx1 positive pancreatic progenitors is required for development of both the pancreatic exocrine and endocrine compartments[20]. Here, we find that Dicer function remains important throughout pancreatic development and plays a role in regulating acinar identity and viability. Our model utilizes a driver line that initiates Dicer recombination in the developing pancreas at a later embryonic stage than the Cre driver used by Lynn and colleagues[21]. We observe minimal gross effects on pancreatic development, suggesting stage sensitive requirements for miRNA processing on establishing and expanding exocrine and endocrine progenitors. However, the acinar cells that do develop are unstable both in regards to their terminal differentiation and viability. Elements of acinar identity are downregulated, and cell stress and apoptosis are both increased. Therefore, competent miRNA processing appears to remain important even in the mature exocrine compartment.

Because Dicer deletion in this model permits pancreatic development we were able to explore the role of Dicer function in Kras mediated PDA development. Mouse models have revealed that oncogenic Kras can act as a “master regulator” of PDA development, establishing lineages that can give rise to PanINs and PDA and remaining critical for progression[1], [2], [3]. Considerable evidence suggests that acinar cells can give rise to PanINs by undergoing ADM, a process during which acinar cells lose terminal differentiation at the expense of a de-differentiated, duct like state[4], [5], [6], [7]. Kras driven ADM occurs gradually, but can be accelerated by compromising acinar differentiation. Insults such as pancreatitis, which causes regeneration associated dedifferentiation[6], [45], activation of progenitor associated pathways (e.g. Notch[24]), and inactivating genes that maintain the acinar state[28], [29], [46] all dramatically accelerate Kras driven ADM and PanIN development. Therefore, loss of acinar identity may be a key barrier to Kras dependent specification of acinar derived PDA precursors. Our data suggest that loss of Dicer function removes the differentiation barrier for Kras driven ADM. ADM occurs at 3 weeks in *Kras; Dicer^Homo^* mice, a time point when the consequences on exocrine differentiation and morphology due to mutant Kras alone are minimal. While Dicer loss compromises acinar differentiation, it does not appear singly sufficient to induce ductal reprogramming, indicating synergy between Kras and Dicer loss to drive ADM. Supporting this role for the miRNA pathway in maintaining acinar differentiation, Prevot and colleagues [47] have recently also shown that Dicer deficient acini display compromised expression of acinar genes and upregulated expression of genes involved in liver development, including the transcription factor HNF6/onecut1. Indeed, we find HNF6/Onecut1 to be highly expressed in sorted acinar cells from caerulein treated *Kras; Dicer^Homo^* versus *Kras; Dicer^Het^* mice (Table S3), suggesting that inappropriate HNF6/Onecut1 expression may also be involved in Kras driven ADM.

Despite inducing unconstrained Kras driven ADM, Dicer loss neither accelerates PanIN nor PDA development. This disconnect is potentially due to increased apoptosis, and thus selection against Dicer deleted cells, demonstrating that maintenance of appropriate levels of miRNA biogenesis is required for viability during Kras driven ADM. These opposing roles, pro-tumorigenic de-differentiation on one hand, cell death on the other, suggest checkpoints might be in place to eliminate miRNA deficient cells undergoing potentially deleterious, transformation sensitive de-differentiation. Supporting this notion, a similar pattern of compromised differentiation in the setting of increased apoptosis has been observed in other Dicer loss of function models. In the liver, for example, Dicer loss leads to the inappropriate activation of pro-proliferative genes expressed in fetal liver development, accompanied by overwhelming levels of cell death[33]. Synthetic lethality between pro-oncogenic signaling, namely tumor suppressor loss, and Dicer function has also been observed in retinoblastoma (Rb), p53, Dicer null retinal progenitor cells[48]. This lethal interaction resembles the synergistic cell death we observe between loss of Dicer function and mutant Kras during unconstrained ADM. Increased cell death also corresponds to decreased expression of genes that have been experimentally validated to support viability during Kras driven PanIN development and in PDA cells, such as Nupr1 and AGR2[35], [36], [37], [49]. If Dicer dependent regulation of these genes depends on specific miRNAs throughout PDA development, targeting those miRNAs directly could also be a route to undermining PDA viability.

Dicer dependent cell death during Kras driven ADM suggests that pancreatic cells exhibit similar negative selection against Dicer loss similar to that seen in a number of tumor models, [13], [14], [15], [48], [50]. In line with this observation, 2 of 3 cell lines derived from PDA in *Kras; Dicer^Homo^* mice maintained at least one intact Dicer allele. Surprisingly though, one of the 3 cell lines derived from PDA in *Kras; Dicer^Homo^* mice displayed recombination of both Dicer alleles and appears to have resulted from a tumor that developed in the absence of Dicer. Processing of some miRNAs has been shown to occur in a Dicer independent fashion, including miR-451[51], [52], a miRNA that has been noted to be abundant in PDA fine needle aspirate samples[53]. Although beyond the scope of this study, compensation by Dicer independent miRNAs could possibly be involved in maintaining viability in the absence of Dicer function. The invasive histology of PDA from *Kras; Dicer^Homo^* mice appeared similar, transformation without Dicer occurs at the expense of some pro-tumorigenic cell biology, as observed in clonally selected Dicer null sarcomas[50]. Cells possessing one Dicer allele and intermediate Dicer expression proliferated faster, and grew larger tumors when transplanted into immune deficient mice, compared to both Dicer deficient cells and cells retaining both Dicer copies. Therefore, PDA, and other mutant Kras driven tumors[12], may depend on maintaining miRNA processing above a critical threshold required for viability and proliferation. While monoallelic Dicer deletions have been reported[13], and variable Dicer levels have recently been observed in PDA cell lines[54], extensive characterization of Dicer and components of the miRNA biogenesis machinery in PDA has not been performed.

In conclusion, we establish intact miRNA processing as an important mediator of Kras driven PDA development. We find that intact Dicer function constrains Kras driven ADM by maintaining acinar differentiation but is also required for cell viability during Kras driven metaplasia. Surprisingly though, mouse PDA can develop without Dicer. Future work understanding the relationship between thresholds of miRNA processing and differentiation and viability could help better define targets for PDA therapy.

## Materials and Methods

### Mouse Lines

Experimental animals were generated by intercrossing mice bearing *Pdx-Cre^Late^* [21], *LSL-Kras^G12D^* [1], *Dicer^flox^* [33] and R26R-EYFP[6] alleles. All mouse experiments were performed under the approval of the University of California, San Francisco Institutional Care and Use of Animals Committee (IACUC, Institutional PHS Assurance #A3400-01), and all efforts were made to minimize animal suffering.

### Immunohistochemistry and Immunofluorescence

Pancreata were fixed overnight in zinc-containing neutral-buffered formalin (Anatech LTD), embedded in paraffin, cut into 5-µm-thick sections, and mounted on Superfrost Plus slides (Fisher Scientific). Sections were subjected to hematoxylin and eosin (H&E), immunohistochemical, and immunofluorescent staining as described[6]. The following primary antibodies were used: rabbit anti-amylase (1∶300; Sigma), rat anti-CK19 (TROMAIII, 1∶200 dilution; developed by Dr. Rolf Kemler [Max-Planck Institute of Immunobiology, Freiburg, Germany] and obtained from the Hybridoma Bank at the University of Iowa), goat anti-clusterin (1∶200; Santa Cruz), rabbit anti-Sox9 (1∶1000; Chemicon), chicken anti-GFP (1∶200; Abcam), biotinylated anti-Cd49f (1∶50 eBioscience), and biotinylated anti-CD133 (1∶50, eBioscience). For immunohistochemistry, biotinylated secondary antibodies were used at a 1∶200 dilution and peroxidase conjugation was performed with the streptavidin based ABC kit (Vector labs). 3-3-Diaminobenzidine tetrahydrochloride (Vector Labs) was used as a chromogen. Bright-field images were acquired using a Zeiss Axio Imager D1 scope. For immunofluorescence detection of primary antibodies, appropriate Alexafluor conjugated secondary antibodies were used at a 1∶200 dilution. Confocal images were collected on a Leica SP5 microscope. Alcian blue staining was performed as described [6], and quantified by collecting 100X images encompassing at least 1 complete tissue section from each mouse and scoring the number of Alcian Blue positive lesions per mm^2^ of total pancreatic area (measured by outlining pancreatic tissue with the Axiovision software package (Zeiss)).

### TUNEL Assay

TUNEL staining was performed with the Apoptag Fluorescein in situ Apoptosis Detection Kit (Chemicon) according to manufacturer’s instructions. The number of TUNEL+ cells per unit YFP+ area were quantified by imaging at least 1 entire tissue section at 100X on an InCell image automated microscope (GE).

### Cell Sorting

Single cell suspensions of 3 week old pancreata were generated with a modified version of a protocol established by Sugiyama and colleagues, 2007 [26]. Briefly, pancreatic lymph nodes were removed, and pancreata were minced and sequentially incubated with collagenase D (1 mg/ml in Hanks buffered saline solution, Roche), trypsin (0.05%, Invitrogen), and dispase (2 U/ml, Invitrogen). Dnase1 (100 ug/ml, Sigma) was added during all enzyme incubations. Cells were washed with PBS between the collagenase and trypsin steps, and with FACs buffer (2% FBS, 10 mM EGTA, in PBS) between the trypsin and dispase steps. Suspensions were then filtered through 40 uM mesh, subjected to FC block (1∶200), and incubated with PE-conjugated anti-Cd49f (1∶50, Ebioscience), PE-CY7-conjugated anti-CD45 (1∶500, Ebioscience), and biotin conjugated anti-CD133 antibodies (1∶50, Ebioscience). Streptavidin conjugated to APC was then applied to detect CD133 positive cells (1∶100, Ebisocience). Cells were washed with FACs buffer between block, primary and secondary antibody incubations. DAPI (300 nM) was used as a live cell marker. Cell sorting was performed on a FacsARIAII (Becton Dickinson).

### RT-PCR

RNA was extracted from sorted cells using the RNAqueous RNA extraction kit (Ambion) according to modified manufacturer’s instructions to include small RNA fractions, and from tissue with the RNEasy kit (Qiagen) as described [6]. cDNA was synthesized using Superscript II Reverse Transcriptase (Invitrogen). Taqman RT-PCR was performed using inventoried probes for mouse *Amylase 2 (*Mm02342486_mH), *Mist1* (Mm00487695_m1), *CK19 (*Mm00492980_m1), *Sox9 (*Mm00448840_m1), *Hes1 (*Mm01342805_m1), *Hey1 (*Mm00468865_m1), *Hey2 (*Mm00469280_m1), *HeyL (*Mm00516555_m1*)* (Applied Biosystems), Nupr1 (Mm00498104_m1), Reg3b (Mm00440616_g1), ITIH4 Mm00497648_m1), and Agr2 (Mm00507853_m1). Expression levels were normalized using a custom primer/probe set for *Cyclophilin* (generously provided by the Genome Analysis Core at the UCSF Helen Diller Family Comprehensive Cancer Center: Primer 1: GGCCGATGACGAGCCC, Primer 2: TGTCTTTGGAACTTTGTCTGCAA, Probe: TGGGCCGCGTCTCCTTCGA). RT-PCR for Dicer was performed using SYBR GREEN master mix (Applied Biosystems) with primers specifically designed to detect deletion of the conditional Dicer allele [33]. Dicer expression was normalized to Gus levels detected by RT-PCR with SYBR GREEN using primers designed by Heiser and colleagues (2008)[21]. miRNA RT-PCR was performed with the Taqman miRNA reverse transcription kit (Applied Bisosystems) and inventoried assays for mouse miR-141 (000463), miR-216a 002220), miR-16 (000391), miR-107 (000443), and miR-191 (002299). miRNA expression was normalized to SnoRNA-202 levels (Applied Biosystems, 001232)

### Caerulein Treatment

*PdxCre^Late^; LSL-Kras^G12D^; Dicer^flox/+^* and *PdxCre^Late^; LSL-Kras^G12D^; Dicer^flox/flox^*; mice (both with and without the R26-EYFP allele) were treated with caerulein as described [6], with 2 sets of 6 hourly i.p. caerulein injections (American Peptide Company, 50 µg/kg) on alternating days separated by 24 hours. The final day of caerulein injection was considered day 0.

### RNA Deep Sequencing

500 ng of total RNA isolated from sorted pancreatic cells was used to generate libraries with the Illumina TruSeq RNA sample preparation kit. Each library was diluted to approximately 10 pM prior to loading and sequenced using a Hi-seq 2000 instrument generating 50 base pair reads. Library preparation and sequencing was performed by the Duke IGSP Genome Sequencing and Analysis Core. The 50 bp long reads were mapped to the mouse genome (NCBI37/mm9) and exon-exon junctions using TopHat [55] version 1.3.1 with default parameters. Genome information was downloaded from the UCSC Genome Browser [56]. We filtered TopHat alignments and kept reads (and their locations) that could be mapped uniquely. Per gene, we extracted read counts by using samtools [57] version 0.1.13. Read counts were normalized to the length of the gene and the total number of mapped reads to obtain a standardized count (RPKM, reads per kilobase of exon model per million mapped reads). Due to the lack of replicates, a Fisher’s Exact Test was used to search for differentially expressed genes. P-values were calculated by comparing the normalized expression level of each gene to the normalized expression level of two housekeeping genes (Gusb, Rpl23). Complete data set is included as Table S3.

### Establishment of PDA Cell Lines and Subcutaneous Tumor Growth Assays

PDA bearing mice we sacrificed and fibrotic tumor areas were removed, minced with scissors, and sequentially digested with collagenase D, trypsin, and dispase as described in the cell sorting methods. Suspensions were filtered through 40 uM mesh and both trapped chunks and flow through were plated on collagen I coated plates (BD). Cells were passed until morphologically clear from fibroblasts and maintained on collagen I coated plates in DMEM containing 10%FBS and penicillin and streptomycin. For subcutaneous tumor growth assays, 5×10^5^ tumor cells resuspended in PBS were mixed 1∶1 with growth factor reduced matrigel (BD) and injected subcutaneously on the back of 6 week old immunodeficient NOD/SCID/gamma null mice. Tumor volume was estimated with calipers using the formula 0.5(Length*(Width)^2^). Tumors were removed when they reached ∼500 mm^3^.

## Supporting Information

Figure S1 **Deletion of Dicer with** ***Pdx1-Cre^Late^*** **permits pancreatic development. (A–D)** Grossly normal pancreas histology in *Dicer^Het^* (A), *Dicer^Homo^* (B), *Kras; Dicer^Het^* (C), and *Kras; Dicer^Homo^* (D) mice at p0. Scale bar 100 μM. **(C, D)**. RT-PCR reveals efficient pancreatic Dicer deletion in *Dicer^Homo^* versus *Dicer^Het^* mice at p0. Mean ± SD. n = 3. E. Reduced Dicer expression at p0 in RNA extracted from *Dicer^Het^* and *Dicer^Homo^* pancreas. Mean ± SD, n = 3.
(TIF)

Figure S2 **Acinar morphology in 3 weeks old** ***Dicer^Het^*** **and** ***Dicer^Homo^*** **pancreas. (A)** Normal acinar morphology and amylase distribution in 3 weeks old *Dicer^Het^* mice. **(B)** Disturbed acinar morphology and fragmented amylase positive structures (arrowheads) in 3 weeks old *Dicer^Homo^* mice. Images are magnified regions from Figure 1B. Note the absence of CK19 expression in *Dicer^Homo^* acinar cells.
(TIF)

Figure S3 **Loss of amylase and increased CK19 expression in mouse ADM. (A–D)** Immunofluorescence staining of YFP, Amylase, and CK19 in a 3-weeks old *Kras; Dicer^Het^; YFP* mouse. Arrowhead indicates a YFP+ structure developing ductal morphology with a region of low amylase and high CK19 expression**.**
(TIF)

Figure S4 **FACS enrichment of pancreatic acinar and ductal compartments. (A)** Representative flow cytometry plot of a pancreas from an adult, 6 week old mouse, dissociated into a single cell suspension. Viable, non-hematopoietic cells (DAPI-CD45-, left panel) were gated and further analyzed for expression of CD49f and CD133 (right panel). **(B, C)** CD49f and CD133 staining in 6 week old mice. Scale bar 100 μM. **(D)** RT-PCR analysis of acinar and ductal markers from CD49f+CD133- and double positive CD49f+CD133+ cells. **(E)** Analysis of Notch effectors in sorted CD49f+CD133- and double positive CD49f+CD133+ cells. Mean ± SD. n = 3.
(TIF)

Figure S5 **Loss of YFP positive cells at 9 weeks in** ***Kras; Dicer^Homo^; YFP*** **mice. (A)** Widespread YFP expression in 9 weeks old *Dicer^Het^; Kras; YFP* mice. **(B)** Reduced and heterogeneous YFP expression in a 9 weeks old *Dicer^Homo^; Kras; YFP* mouse.
(TIF)

Figure S6 **Isolating acinar cells for determination of Dicer dependent genes during Kras driven ADM.** **(A,B).** H&E staining of *Kras; Dicer^Het^* (A), and *Kras; Dicer^Homo^* (B) pancreas 2 days after caerulein treatment. Scale bars 100 μM **(C,D).** H&E staining of *Kras; Dicer^Het^* (C), and *Kras; Dicer^Homo^* (D) pancreas 21 days after caerulein treatment. Scale bars 100 μM **(E).** Representative sorting profiles of *Kras; Dicer^Het^*; *EYFP* and *Kras; DicerHomo; YFP* 2 days after caerulein. Gated CD49f+, CD133- populations in right panels were collected for analysis.
(TIF)

Table S1 (XLSX)

Table S2 (XLSX)

Table S3 (XLS)
