# Supplementary figures and images for: Dicer Regulates Differentiation and Viability during Mouse Pancreatic Cancer Initiation
Source: PLoS One. 2014 May 1;9(5):e95486. doi: 10.1371/journal.pone.0095486 (PMC4006805; doi:10.1371/journal.pone.0095486)

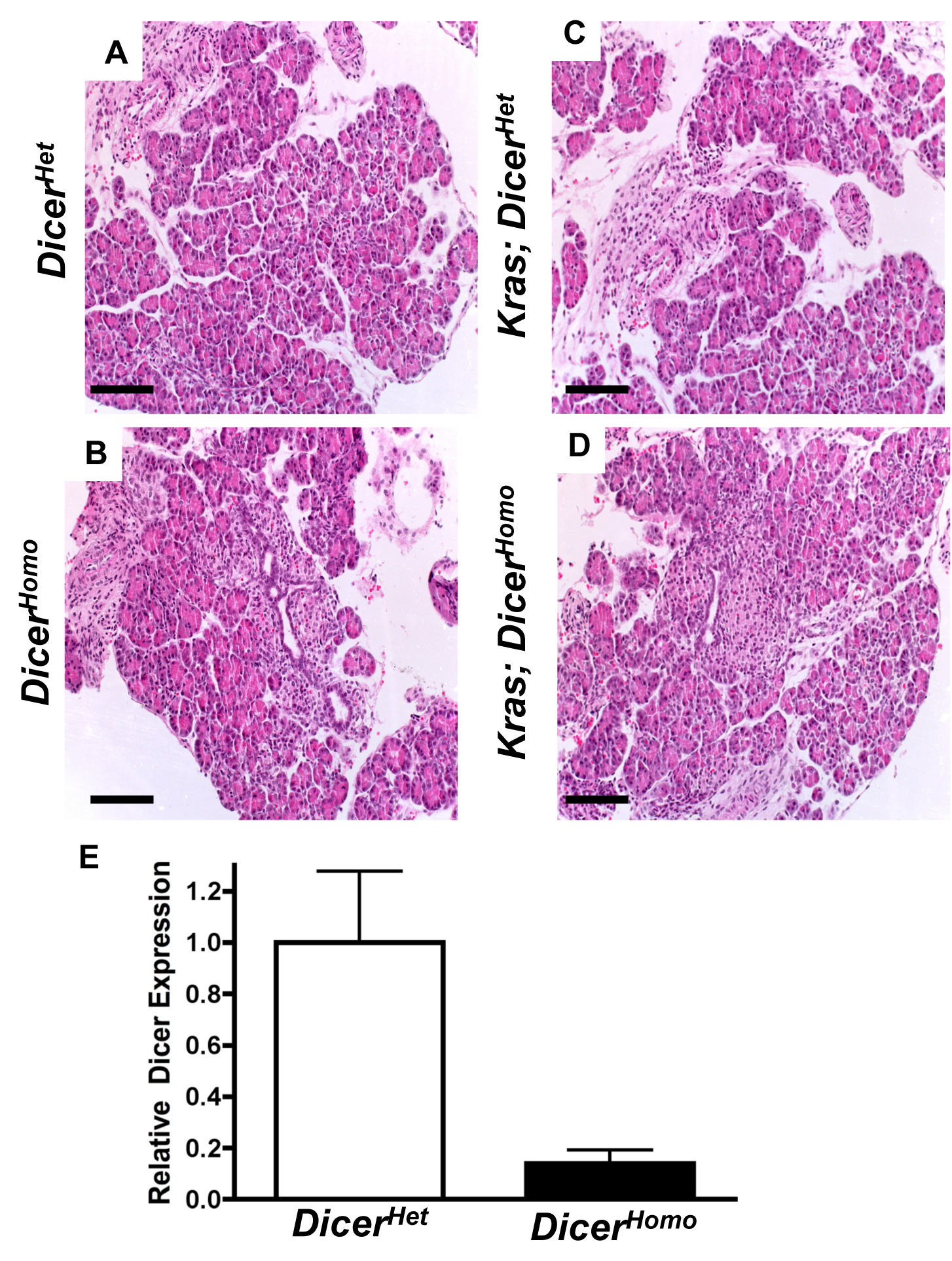

Supplement: Figure S1 — Deletion of Dicer with Pdx1-CreLate permits pancreatic development. (A–D) Grossly normal pancreas histology in DicerHet (A), DicerHomo (B), Kras; DicerHet (C), and Kras; DicerHomo (D) mice at p0. Scale bar 100 μM. (C, D). RT-PCR reveals efficient pancreatic Dicer deletion in DicerHomo versus DicerHet mice at p0. Mean ± SD. n = 3. E. Reduced Dicer expression at p0 in RNA extracted from DicerHet and DicerHomo pancreas. Mean ± SD, n = 3. (TIF) [file pone.0095486.s001.tif]

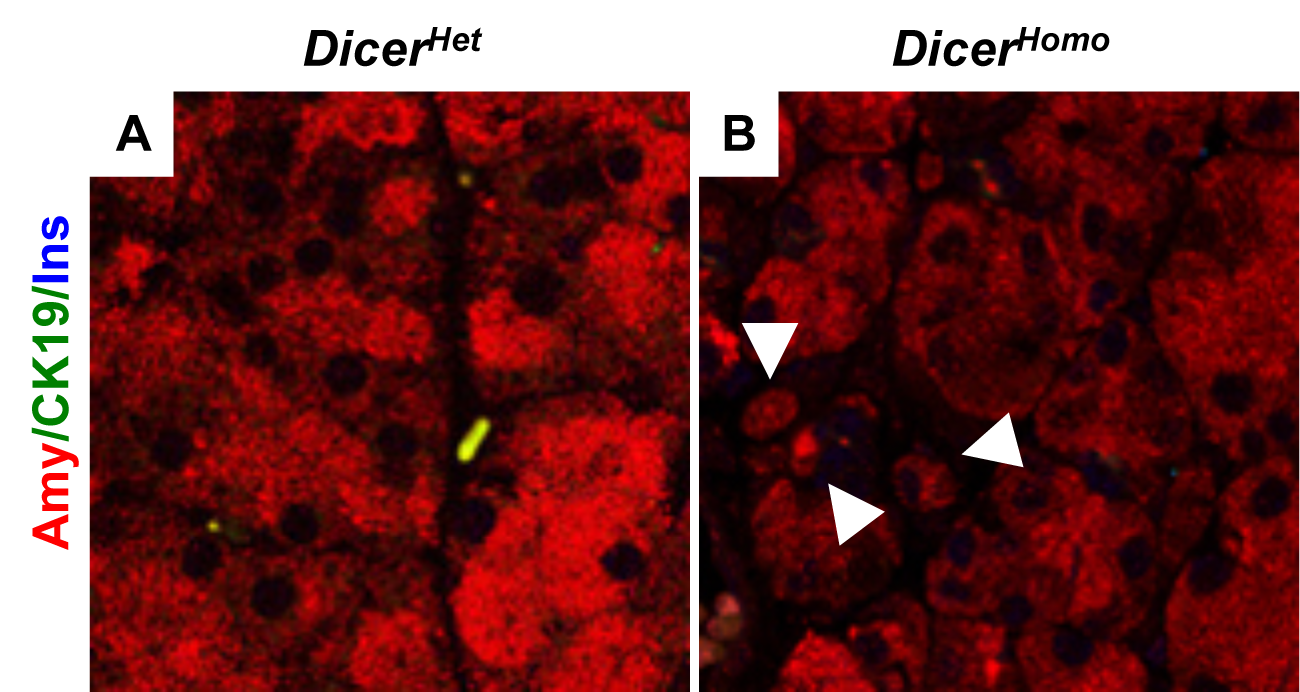

Supplement: Figure S2 — Acinar morphology in 3 weeks old DicerHet and DicerHomo pancreas. (A) Normal acinar morphology and amylase distribution in 3 weeks old DicerHet mice. (B) Disturbed acinar morphology and fragmented amylase positive structures (arrowheads) in 3 weeks old DicerHomo mice. Images are magnified regions from Figure 1B. Note the absence of CK19 expression in DicerHomo acinar cells. (TIF) [file pone.0095486.s002.tif]

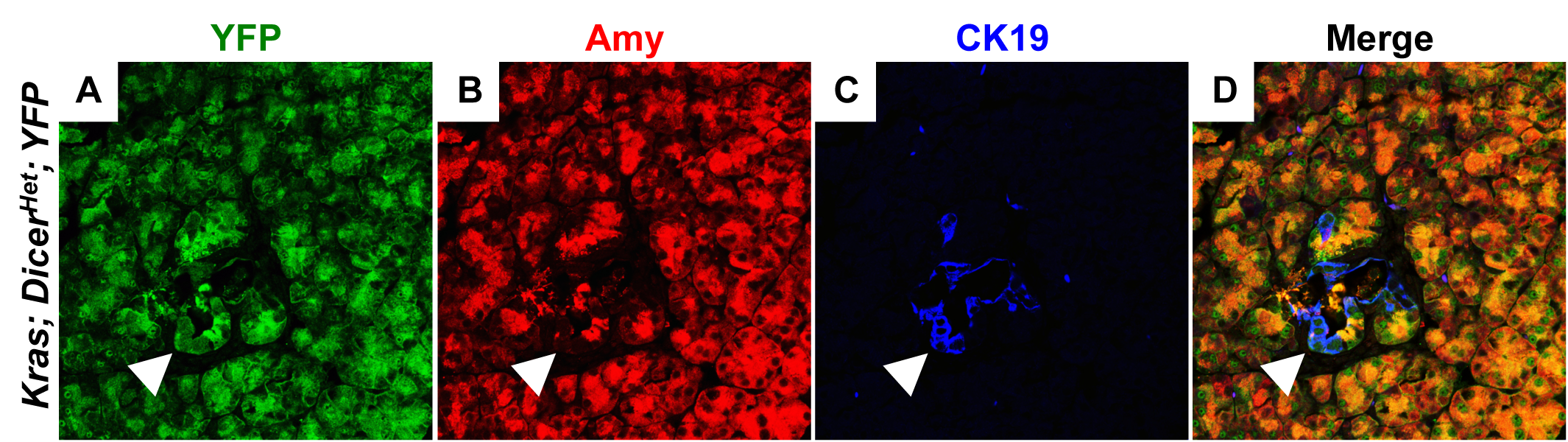

Supplement: Figure S3 — Loss of amylase and increased CK19 expression in mouse ADM. (A–D) Immunofluorescence staining of YFP, Amylase, and CK19 in a 3-weeks old Kras; DicerHet; YFP mouse. Arrowhead indicates a YFP+ structure developing ductal morphology with a region of low amylase and high CK19 expression. (TIF) [file pone.0095486.s003.tif]

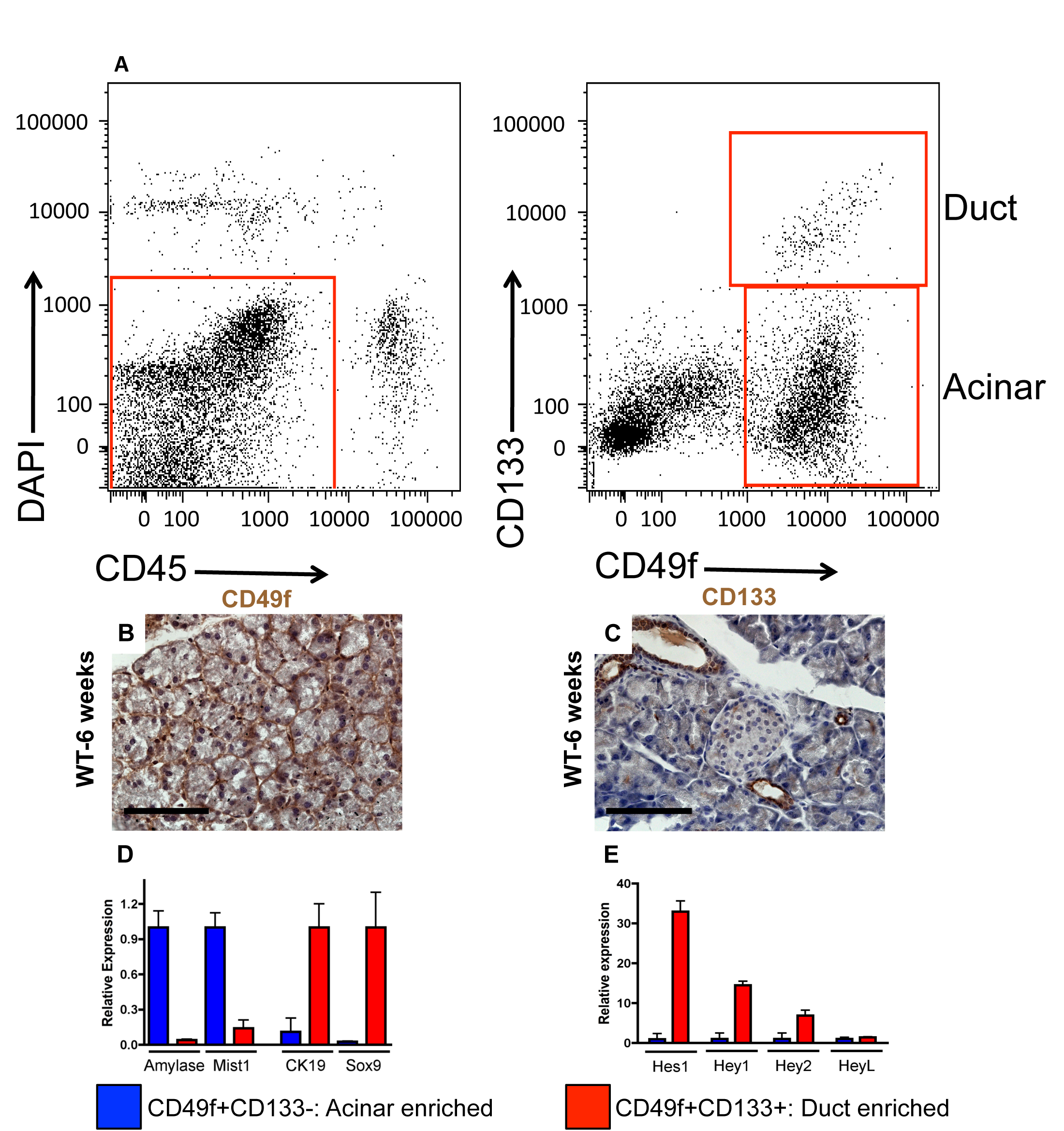

Supplement: Figure S4 — FACS enrichment of pancreatic acinar and ductal compartments. (A) Representative flow cytometry plot of a pancreas from an adult, 6 week old mouse, dissociated into a single cell suspension. Viable, non-hematopoietic cells (DAPI-CD45-, left panel) were gated and further analyzed for expression of CD49f and CD133 (right panel). (B, C) CD49f and CD133 staining in 6 week old mice. Scale bar 100 μM. (D) RT-PCR analysis of acinar and ductal markers from CD49f+CD133- and double positive CD49f+CD133+ cells. (E) Analysis of Notch effectors in sorted CD49f+CD133- and double positive CD49f+CD133+ cells. Mean ± SD. n = 3. (TIF) [file pone.0095486.s004.tif]

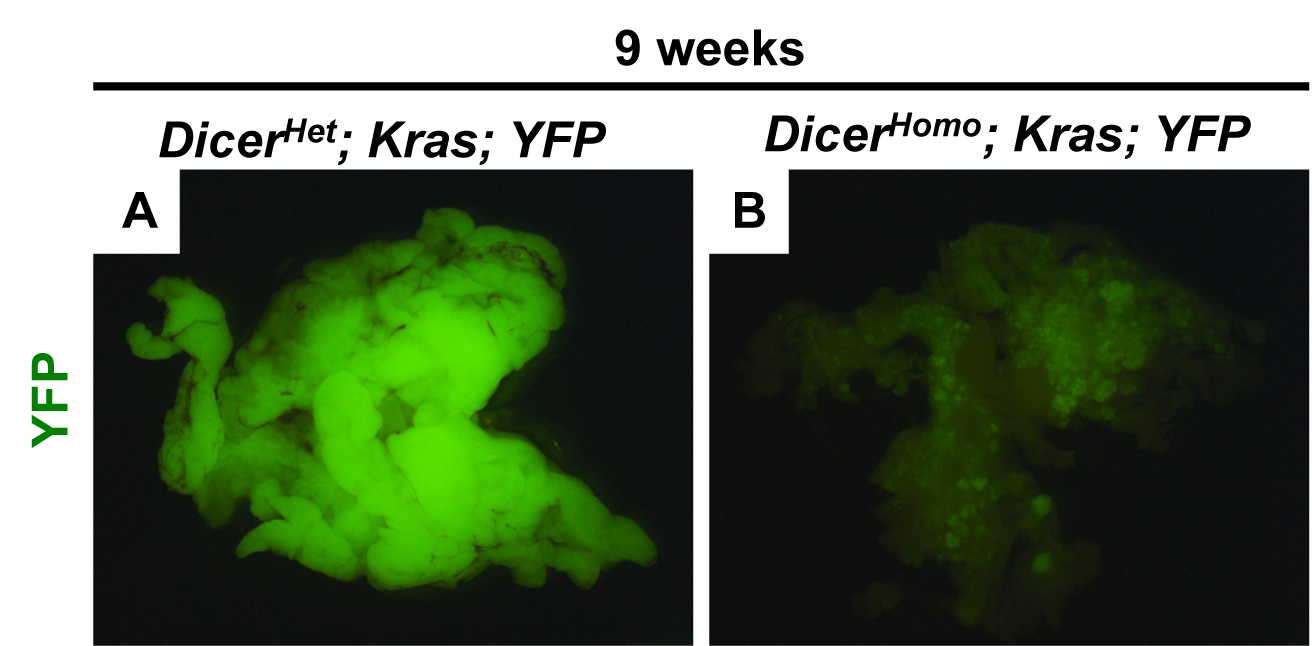

Supplement: Figure S5 — Loss of YFP positive cells at 9 weeks in Kras; DicerHomo; YFP mice. (A) Widespread YFP expression in 9 weeks old DicerHet; Kras; YFP mice. (B) Reduced and heterogeneous YFP expression in a 9 weeks old DicerHomo; Kras; YFP mouse. (TIF) [file pone.0095486.s005.tif]

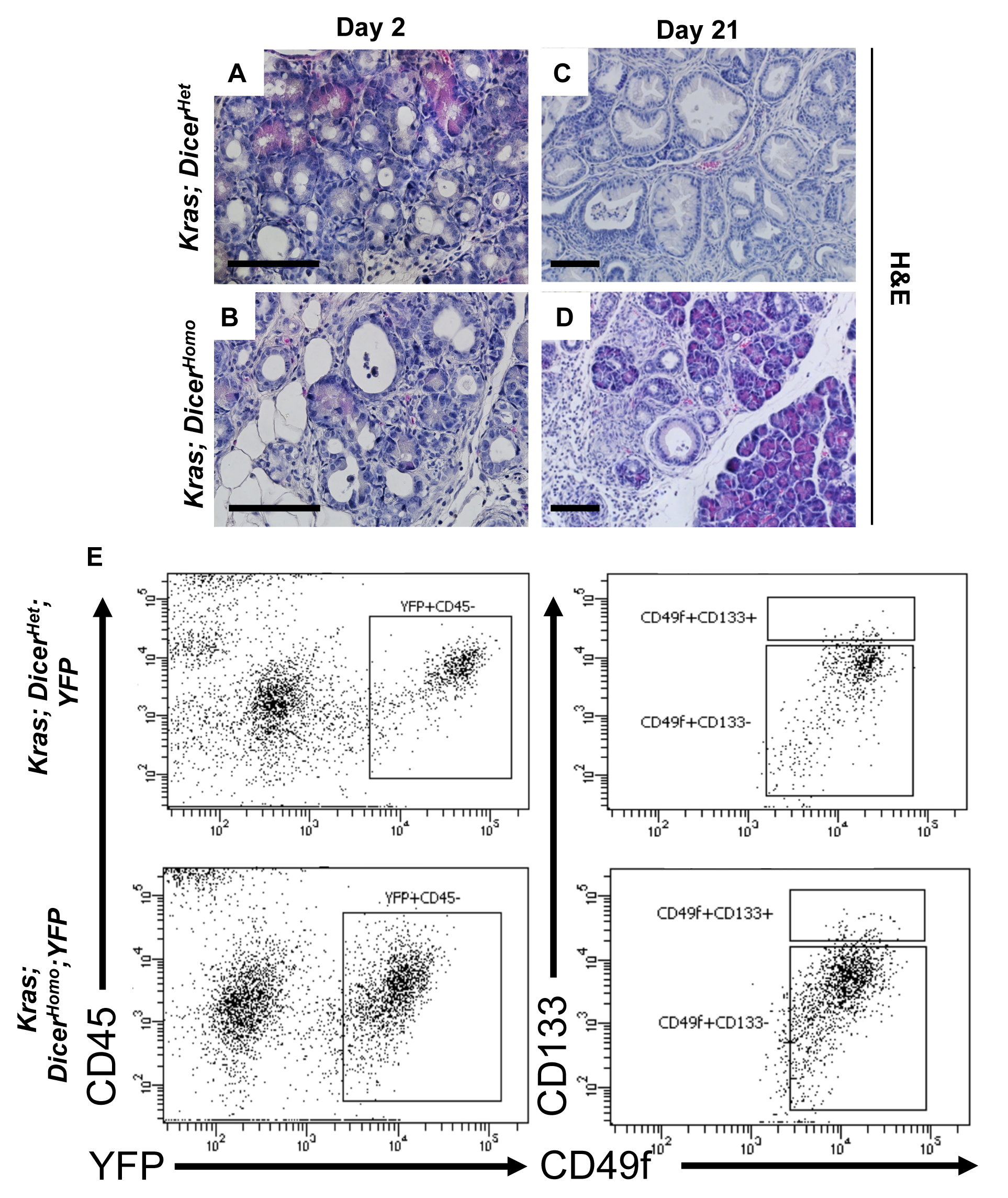

Supplement: Figure S6 — Isolating acinar cells for determination of Dicer dependent genes during Kras driven ADM. (A,B). H&E staining of Kras; DicerHet (A), and Kras; DicerHomo (B) pancreas 2 days after caerulein treatment. Scale bars 100 μM (C,D). H&E staining of Kras; DicerHet (C), and Kras; DicerHomo (D) pancreas 21 days after caerulein treatment. Scale bars 100 μM (E). Representative sorting profiles of Kras; DicerHet; EYFP and Kras; DicerHomo; YFP 2 days after caerulein. Gated CD49f+, CD133- populations in right panels were collected for analysis. (TIF) [file pone.0095486.s006.tif]
